# Identification of a robust subpathway-based signature for acute myeloid leukemia prognosis using an miRNA integrated strategy

**DOI:** 10.1371/journal.pone.0194245

**Published:** 2018-03-23

**Authors:** Huijuan Chang, Qiuying Gao, Wei Ding, Xueqin Qing

**Affiliations:** 1 Department of Pneumology, Children’s Hospital Affiliated to Zhengzhou University, Henan Children’s Hospital, Zhengzhou Children’s Hospital, Zhengzhou, Henan, China; 2 Deparment of Haematology, Shaanxi Provincial People’s Hospital, Xi’an, Shanxi, China; 3 Department of Gastroenterology, 18 Bureau Gaobeidian Hospital, Gaobeidian, Hebei, China; 4 Department of Pediatric, Shanghai General Hospital, Shanghai, China; College of Bioinformatics Science and Technology, CHINA

## Abstract

Acute myeloid leukemia (AML) is a heterogeneous disease, and survival signatures are urgently needed to better monitor treatment. MiRNAs displayed vital regulatory roles on target genes, which was necessary involved in the complex disease. We therefore examined the expression levels of miRNAs and genes to identify robust signatures for survival benefit analyses. First, we reconstructed subpathway graphs by embedding miRNA components that were derived from low-throughput miRNA-gene interactions. Then, we randomly divided the data sets from The Cancer Genome Atlas (TCGA) into training and testing sets, and further formed 100 subsets based on the training set. Using each subset, we identified survival-related miRNAs and genes, and identified survival subpathways based on the reconstructed subpathway graphs. After statistical analyses of these survival subpathways, the most robust subpathways with the top three ranks were identified, and risk scores were calculated based on these robust subpathways for AML patient prognoses. Among these robust subpathways, three representative subpathways, path: 05200_10 from Pathways in cancer, path: 04110_20 from Cell cycle, and path: 04510_8 from Focal adhesion, were significantly associated with patient survival in the TCGA training and testing sets based on subpathway risk scores. In conclusion, we performed integrated analyses of miRNAs and genes to identify robust prognostic subpathways, and calculated subpathway risk scores to characterize AML patient survival.

## Introduction

Acute myeloid leukemia (AML) is a biological heterogeneous disease, and its major characteristic is clonal proliferation of immature myeloid cells [1]. Currently, chemotherapy-based regimens fail to cure the majority of AML patients. And stem cell transplantation is a common treatment choice for these patients [2, 3]. However, the stem cell transplantation is not an option for all AML patients, which further illustrates the genetic heterogeneous nature of this disease. The present staging system is the common patient prognosis index in clinical analysis, and the patients with late stage disease usually display worse prognoses [1, 4]. However, in most cases, patients with the same clinical stage may experience totally different clinical outcomes, especially for the patients with late stage. In addition, some patients with AML develop early metastasis, while some may not develop this complication. Therefore, there is a critical need to develop a novel strategy for exploring biological mechanisms involved in AML to identify markers for clinical characterization of this disease.

High-throughput gene expression levels can be utilized to study the biological heterogeneity issues of complex disease. For certain disorders, studies have identified different genes as risk markers, and have confirmed the usefulness of using these genes in their studies. Studies have reported the statistically significant association of gene expressions with AML. For example, Costa et al. performed the meta-analysis and identified the immunophenotypic markers at the gene expression levels. As a result, twelve antigens exhibited a negative impact with AML prognosis, and provided further guidance for new therapeutic targets [5]. By analyzing the available data sets, Huang et al. identified 11 genes as a potential prognostic marker of AML, and demonstrated the good performances for predicting overall survival in the validation cohorts [6]. More importantly, functionally-based analyses could be used for better interpretation of disease formation and progression [7, 8]. At a functional level, different gene signatures may play similar biological roles, with robust common functions. Thus, a number of functionally-based methods have been developed as signatures for better biological interpretations [9–11], and have displayed more robust results than gene-based analyses. Multiple genes involved in the same biological processes often act together during disease formation and progression. In addition, functionally-based analyses can reduce the number of dimensions of variables (from genes to functions), and the representative functions can increase the biological robustness necessary for exploring the heterogeneous diseases.

Through post-transcriptional regulation, microRNAs (miRNAs) can further affect the functional activities by inhibiting the expression levels of messenger RNAs (mRNAs) [12–14]. High-throughput technologies can simultaneously measure the expression levels of multiple genes or miRNAs, and can be used to characterize functional conditions in complex diseases. During disease formation and progression, the dysregulated genes usually interact with each other within biological processes or pathways, and have close regulatory relationships with miRNAs [15, 16]. For AML survival, the study of Shah et al. identified and characterized the NRF2-regulated miRNAs. Their findings have revealed the NRF2 regulation of miR-125B and miR-29B acted to promote AML cell survival, which further indicated the involvement of miRNA-gene regulation in AML prognoses [17]. In addition, by miRNA-gene network analysis, Zhang et al. found novel biological functions and pathways involved in AML survival, and mir-425 and CD44 were both identified as risk factors [18]. However, most function-level methods mentioned above were performed based only on gene expression, and the regulatory roles of miRNAs were not determined.

Studies of biological pathways display an advantage because of their topological structures. Because most pathways contain numerous genes, the concept of a subpathway (pathway region) was defined in previous studies [19, 20]. Containing a smaller scale of components, subpathways provide more information in terms of interpreting functional conditions. Abnormalities of subpathways are associated with the etiology of multiple types of diseases [21], further illustrating the advantages of analyses of subpathways compared to more complex entire pathways. We therefore considered both miRNA expression and the subpathway scale to comprehensively identify functional signatures for AML.

In this study, we performed a robust bioinformatics analysis to determine the expression levels of miRNAs and genes to identify prognostic subpathway-based signatures, and the subpathway contained the miRNA and gene components. First, we obtained the gene-based subpathway graphs and embedded the miRNA molecules into these subpathway graphs by considering experimentally validated miRNA-gene relationships. Then, we randomly divided the total data set from The Cancer Genome Atlas (TCGA) into one training set and one testing set. Based on the training set, we obtained 100 subsets, and then identified survival-related miRNAs and genes using the univariable Cox method. Survival related subpathways were further identified by considering miRNAs and genes in each training subset. Using statistical counting, we finally identified six robust subpathways as a prognostic signature and verified their predictive power using an independent testing set.

## Materials and methods

### Training and testing sets from TCGA

We downloaded and obtained the AML data set, including expression data sets and patient clinical information from TCGA data portal (https://portal.gdc.cancer.gov/), which is an interactive data system for researchers to search, download, upload, and analyze harmonized cancer genomic data sets. For the AML expression data sets, the gene expression was generated using HTseq-FPKM, and the miRNA expression was generated using BCGSC miRNA profiling. We directly obtained the expression values (FPKM values) for each miRNA and gene per AML sample, and the average expression values for an miRNA or gene was calculated for duplicated samples. In addition, we eliminated the samples with survival times < 1 month, because these AML patients might have died for other reasons than the disease [22]. Finally, the expression data sets (including the miRNAs and genes) and clinical information of a total of 112 patients were utilized in this study, and we further randomly divided them into a training set (n = 76) and a testing set (n = 36). These two data sets resulted in the same ratio of good and poor survival samples with median survival times as a cutoff, which was also the same as the original data set. The training set and the testing set were totally independent of each other. We utilized the training set to identify robust survival-related subpathways and the testing set to verify the signature predictive power.

### The miRNA-gene interaction data

The miRNA-gene interactions were obtained from the miRTarBase [23], mir2Disease [24], miRecords [25], and TarBase [26] databases. By integrating these four databases, a total of 55146 miRNA-gene interactions between 1110 miRNAs and 20186 genes were obtained. Among these interactions, 6459 miRNA-gene interactions involving 358 miRNAs and 3452 genes that had been verified by low-throughput experiments were utilized in this study.

### The reconstructed subpathways by embedding miRNAs

First, we utilized the available R package [27] to obtain all biological pathways from the KEGG pathway database, which included 152 metabolic pathways and 191 non-metabolic pathways. Then, we utilized the K-clique method [19] to define and locate subpathway regions based on the distance similarity strategy in each metabolic and non-metabolic pathway, and the default K value (3) was used. After these processes, we obtained the gene-based subpathway regions. Then, by considering the miRNA-gene interactions from the verified data, we further embedded the miRNA components into biological subpathways, and the miRNA that regulated at least one gene within one subpathway was included in the corresponding subpathway. Finally, we considered the scale of the miRNA-embedded subpathway graphs, and the small scale graphs with less than one miRNA or three genes were eliminated in further analyses.

### Identification of survival-related subpathways

Based on the reconstructed subpathways containing both gene and miRNA components, we further identified survival-related subpathways by integrating miRNA and gene levels. First, using the expression data sets derived from TCGA training set, we identified survival miRNAs and genes using the univariable Cox method with a significance value of P < 0.05. Then, we mapped these survival miRNAs and genes into the reconstructed subpathway graphs. To evaluate whether one subpathway was related with patient survival at both the gene and miRNA levels, we utilized the hypergeometric test to evaluate the integrated statistical significance as follows:

$$P=1-\sum_{k=0}^{r_{g}+r_{mir}-1}\frac{\left(\begin{array}{c} t_{g}+t_{mir}\\ k \end{array})(\begin{array}{c} m_{g}+m_{mir}-t_{g}-t_{mir}\\ n_{g}+n_{mir}-k \end{array}\right)}{\left(\begin{array}{c} m_{g}+m_{mir}\\ n_{g}+n_{mir} \end{array}\right)}$$

Where *m_g_* and *m_mir_* were the numbers of all genes and miRNAs in the TCGA data sets, and *n_g_* and *n_mir_* were the numbers of survival genes and miRNAs obtained from univariate Cox analysis, of which *r_g_* genes and *r_mir_* miRNAs were also included in one subpathway graph. And this subpathway graph contained *t_g_* genes and *t_mir_* miRNAs. The subpathways with hypergeometric p-value < 0.05 were regarded as prognostic signatures at the miRNA-gene integrated levels.

### Calculation of single and combined subpathway risk scores

Based on each robust survival subpathway, we calculated the risk score by considering both miRNAs and genes that were involved in the subpathway. The risk score was a linear combination of the expression value for each miRNA and gene multiplied by a weighting value obtained from the univariate Cox analysis using the training set. For multiple subpathways, the combined risk score was also a linear combination of the risk score for these subpathways multiplied by a weighting value calculated by univariate Cox analyses based on the risk score in the training set.

## Results

### Construction of subpathway graphs and utilization of TCGA data sets

We utilized the available R package software to obtain subpathway graphs from the KEGG database by maintaining original topology information, and we embedded the regulatory roles of miRNAs by considering the low-throughput miRNA-gene interactions (see Materials and Methods). As a result, the constructed subpathway graphs contained an average of 21.8 miRNAs and 19.5 genes by the node level, and 29.2 miRNA-gene interactions by the edge level, and a total of 1773 subpathways with at least one miRNA and three genes were finally obtained for the identification of survival subpathways. Compared with entire pathways, the subpathway graphs contained smaller sets of components and smaller regulatory interactions between miRNAs and genes. The detailed information of these subpathways was provided in S1 Table.

**Fig 1:**
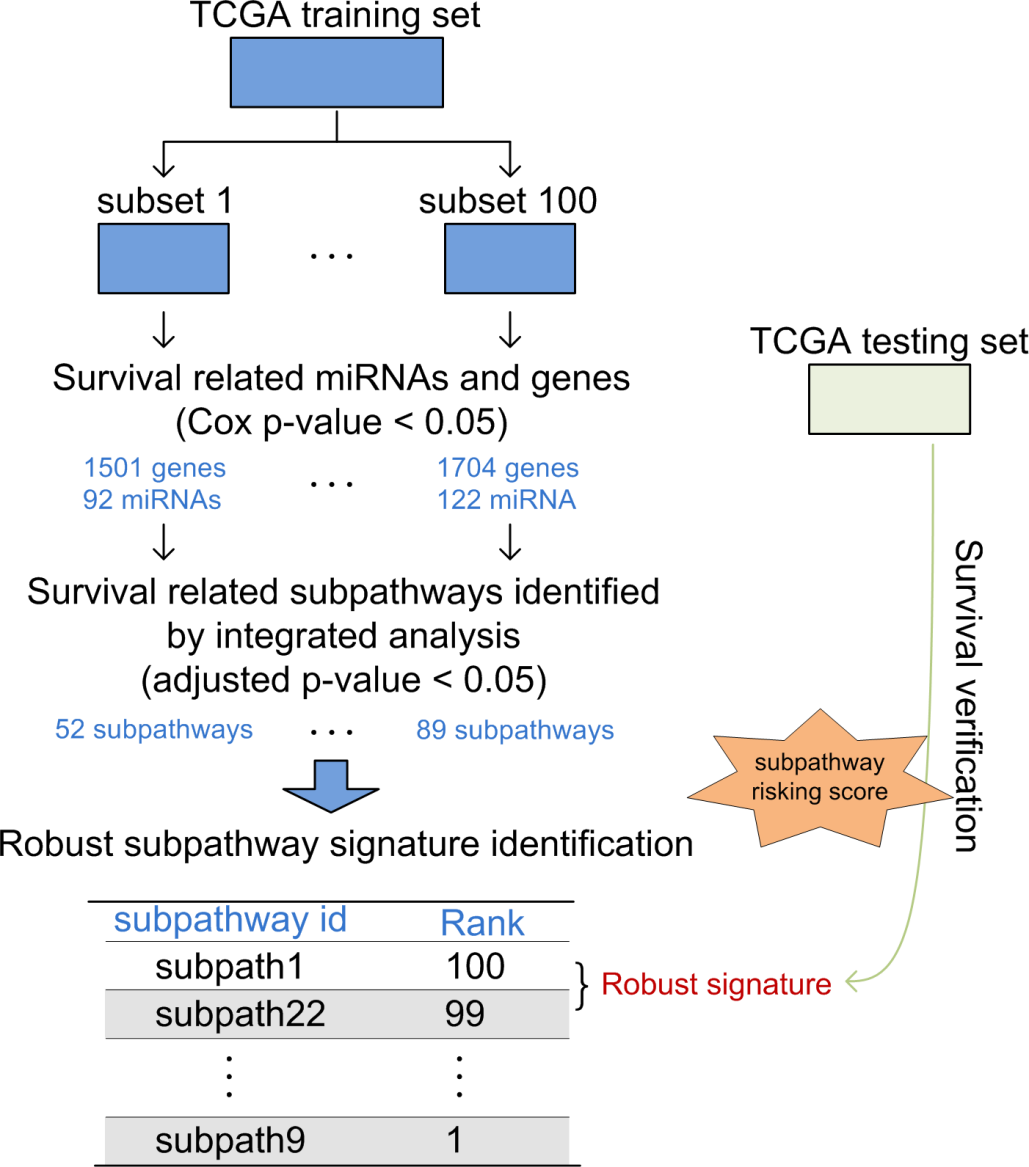
The study design. The training set was used to identify robust prognostic subpathways, and the testing set was used to verify the predictive power of subpathway signatures.

Based on the constructed subpathway graphs involving genes and miRNAs, we performed miRNA-gene integrated analyses to identify robust survival subpathways (Fig 1). First, we randomly divided the total TCGA data set into a training set and a testing set, which contained the same ratio of “good” and “poor” survival samples (median survival time as cutoff). There was no significant difference of patient characteristics between the training and testing data sets (S2 Table). Then, we utilized the training set to perform bioinformatics analyses to identify robust survival subpathways. Finally, we utilized the testing set to verify the predictive performance of the robust subpathway-based signatures.

### Identification of robust survival subpathways

Based on the training set, we performed a bootstrap process to identify robust survival subpathways. First, we randomly selected half of the total training set and formed 100 training subsets (from subsets 1–100). These subsets maintained the same ratio of “good” and “bad” survival samples (median survival time as cut-off) as the total training set. For each training subset, we then performed univariate Cox analyses to identify survival miRNAs and genes with a P-value < 0.05. The survival subpathways were also identified by integrated analyses of the levels of miRNAs and genes using the hypergeometric test (see Materials and Methods). Finally, based on 100 training subsets, we identified 100 survival subpathway lists and obtained a total of survival subpathways by merging all the results. Thus, each survival subpathway included was assigned to a count value from 1–100, and the subpathways with higher values were more robust. As shown in S1 Fig, the subpathway (path: 04662_8) from the B cell receptor signaling pathway displayed a first value (48), which meant that this subpathway was significantly related with survival in a total of 48 training subsets. Moreover, the subpathways from the Pathways in cancer and the T cell receptor signaling pathway displayed high values, and some subpathways from the Cell cycle were also ranked in the top ten. These subpathways were involved in the biological mechanisms of tumors, so further exploration and dissection could provide guidance for AML survival implications. Detailed information of the top 50 robust subpathways is shown in S3 Table.

### Robust subpathways significantly predicted patient clinical outcomes

**Fig 2:**
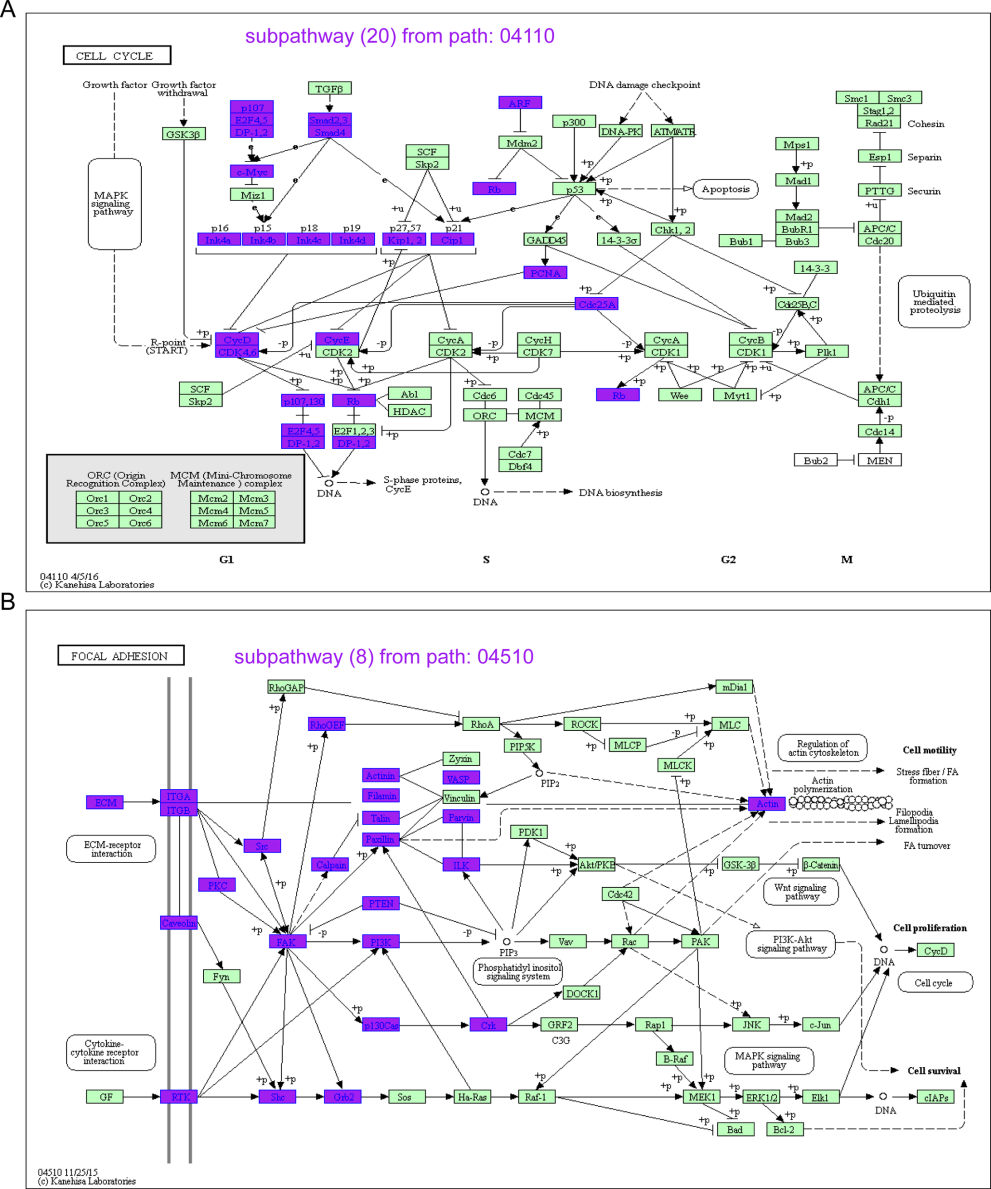
The two subpathways. (**A**) Subpathway_20 from the Cell cycle (path: 04110). (**B**) Subpathway_8 from Focal adhesion (path: 04510).

**Fig 3:**
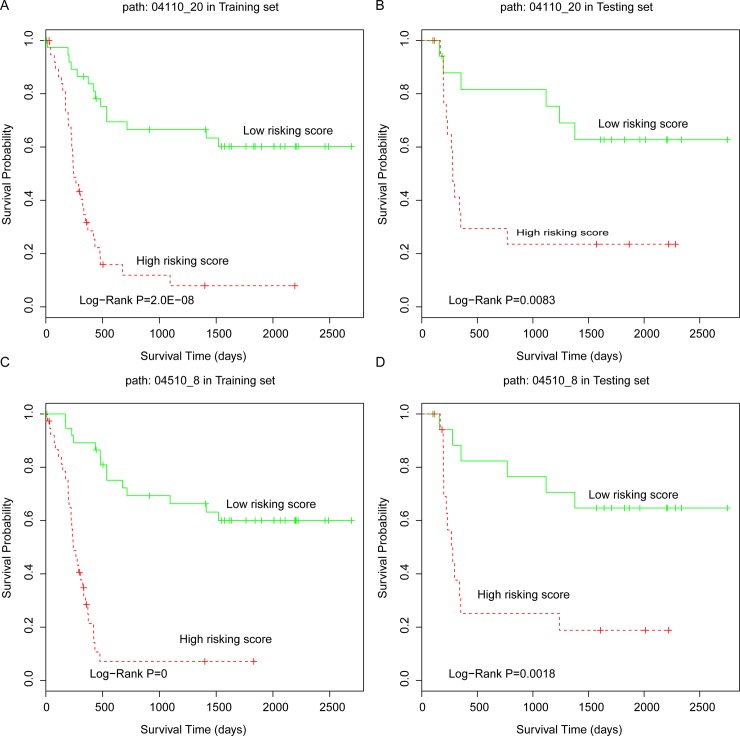
The K-M curves of robust subpathways. (**A**) The 04110_20 in the training set. (**B**) The 04110_20 in the testing set. (**C**) The 04510_8 in the training set. (**D**) The 04510_8 in the testing set. The P-value was calculated using the log-rank test.

Based on each robust subpathway, we calculated the risk score for survival analyses (see Materials and Methods). The risk score simultaneously considered the contributions of miRNAs and genes. Furthermore, the median score was taken as the cutoff for the low risk and high risk group classification. The Kaplan-Meier method was used to generate survival curves and the difference between two survival curves was evaluated by using the log-rank test. All tests were two-tailed, and a value of P < 0.05 was considered to indicate a significant result. Among the robust subpathways with rank value > 43, we observed that three representative subpathways, path: 05200_10 from Pathways in cancer, path: 04110_20 from the Cell cycle, and path: 04510_8 from Focal adhesion were associated with the survival status of AML patients (Fig 2 and S2 Fig). For TCGA training set, the survival P-value of the risk subpathway score for path: 05200_10, path: 04110_20, and path: 04510_8 were 1.0E-08, 2.0E-08, and 0, respectively (Fig 3). Similarly, the risk scores were calculated for the samples in the testing set and two risk groups (high risk and low risk). In accordance with the results from the training set, patients with a high subpathway risk score belonged to the high risk group, and the patients with low subpathway risk scores belonged to the low risk group. The log-rank test showed that there was a significant difference of survival times between the low risk and high risk groups (P = 0.008 for path: 04110_20, see Fig 3B; P = 0.002 for path: 04510_8, see Fig 3D; and P = 0.023 for path: 05200_10, see S2 Fig). Based on the results for these three subpathways, we further defined the combined subpathway risk score for AML survival analyses (see Materials and Methods). For the test set, the combined risk score was also related to the survival status of AML patients with P = 0.021, further showing the predictive performance of the robust survival subpathways.

### Functional analyses of the subpathway signatures

To dissect the functional roles of the subpathway signatures, we first extracted the gene components within these subpathways and performed DAVID functional enrichment analyses. As shown in S4 Table, some signaling terms, including epidermal growth factor receptor signaling pathway (GO: 0007173) and ERBB2 signaling pathway (GO: 0038128), were significantly enriched in the Path: 05200_10 subpathway. Moreover, immune related terms were also included, including leukocyte migration. For the Path: 04110_20 subpathway, some GO terms regarding cell cycle regulation were identified as significant. Similarly, the Path: 04510_8 subpathway was also related with immune, angiogenesis, and cell adhesion, including cell adhesion (GO: 0007155) and cell-substrate adhesion (GO: 0031589). The cell adhesion and cell cycle functions were involved in different types of human tumors.

## Discussion

Previous studies have reported that the gene-based expression signatures derived from different studies shared poor overlaps, which suggested the necessity of a functionally-based analytical strategy. In this study, we identified robust survival subpathways for AML survival analysis using the sample-matched miRNA and gene expression data sets from TCGA database. In the previous studies, the regulatory relations between miRNAs and genes were considered in the AML prognosis analysis [17, 18]. However, in this study, we considered both the expression levels of miRNAs and genes into the survival subpathway identification based on the reconstructed graphs. Overall, this was the first study to identify AML survival signatures at the subpathway level by integrated analyses of both miRNAs and genes.

Many methods have been developed to analyze tumor biological mechanisms at the pathway level. Ooi *et al*. developed a computational approach to identify tumor molecular pathways, and the new driving pathway patterns they found could be used to define clinically relevant subgroups for gastric cancers [10]. Moreover, Huang *et al*. proposed a pathway-based model by including the Cox proportional hazard regression method and further applied this model to predict outcomes for breast cancer patients [11]. However, most current studies performed the function- or pathway-level analyses by considering only gene expression, and did not include the regulatory roles of small non-coding RNAs, for example miRNAs.

Although biological pathways exhibited advantages over Gene Ontology functional terms because of their topology information, the large amount of genes within an entire pathway scale presented challenges for previous analyses. To resolve this issue, the subpathway concept (pathway regions within the entire pathway) was used in a previous study [19]. Compared with the entire pathway, the subpathway provided more precise information for functional enrichment analyses. In addition, studies showed that abnormalities of subpathways, and not the entire pathway, were associated with disease etiology. The subpathway-level analysis method was also applied to explore other biological mechanisms, for example, the actions of drugs [28]. The subpathway concept could therefore be used for identifying AML survival signatures, and the roles of miRNAs should also be considered.

Using the training set, we performed an analysis to identify the most robust survival subpathways, and three representative subpathways were identified, including path: 05200_10 from Pathways in cancer, path: 04110_20 from Cell cycle, and path: 04510_8 from Focal adhesion. Pathways in cancer is a tumor pathways which was associated with multiple of tumor types. Also, the cell cycle arrest was involved in the molecular mechanism of AML cells, which correlated with known responses to therapy [29, 30]. Moreover, the study of Carter et al. fount that the kinase FAK within the focal adhesion pathway regulated leukemia-stromal interactions and supported leukemia cell survival, thus could be a potential therapeutic target in AML [31]. Based on these robust subpathways, we further calculated single and combined risk scores, which combined the Cox coefficient and expression values of all miRNAs and gene components involved in corresponding subpathway graphs. Notably, survival subpathways were identified by considering both survival-related miRNAs and survival-related genes using a hypergeometric test; however, all miRNA and gene components (including significant and insignificant) within the subpathway were included in the calculation of the risk score. The risk score we calculated therefore reflected the overall risk condition at the subpathway level.

We integrated high-throughput miRNAs, mRNA expressions, and pathway structures to systematically identify robust subpathways, and further calculated subpathway-based risk scores for predicting AML patient prognoses. With more sample-matched miRNA and mRNA expression data sets, we further validated the predictive performance of the subpathway-based signatures. Notably, the subpathway signatures we identified contained smaller set of miRNA and gene components than corresponding entire pathways. And the key components within these subpathways also had the potential to be prognostic marker for clinical applications. In the current study, our findings were potentially useful for understanding AML biological events and identifying functional signatures for clinical use.

## Supporting information

S1 Fig The distribution of count values for significant subpathways. (TIF)

S2 Fig (**A**) Subpathway_10 from Pathways in cancer (path: 05200). (**B**) The 05200_10 in the training set. (**C**) The 05200_10 in the testing set.
(TIF)

S1 Table Detailed information of subpathway graphs. (XLS)

S2 Table The clinical features of acute myeloid leukemia patients in The Cancer Genome Atlas training and testing sets. (DOC)

S3 Table Detailed information on the top 50 subpathways. (XLS)

S4 Table Top 30 significant Gene Ontology terms enriched by the gene components within three subpathways. (XLS)
