# Supplementary figures and images for: Identification of a robust subpathway-based signature for acute myeloid leukemia prognosis using an miRNA integrated strategy
Source: PLoS One. 2018 Mar 23;13(3):e0194245. doi: 10.1371/journal.pone.0194245 (PMC5865743; doi:10.1371/journal.pone.0194245)

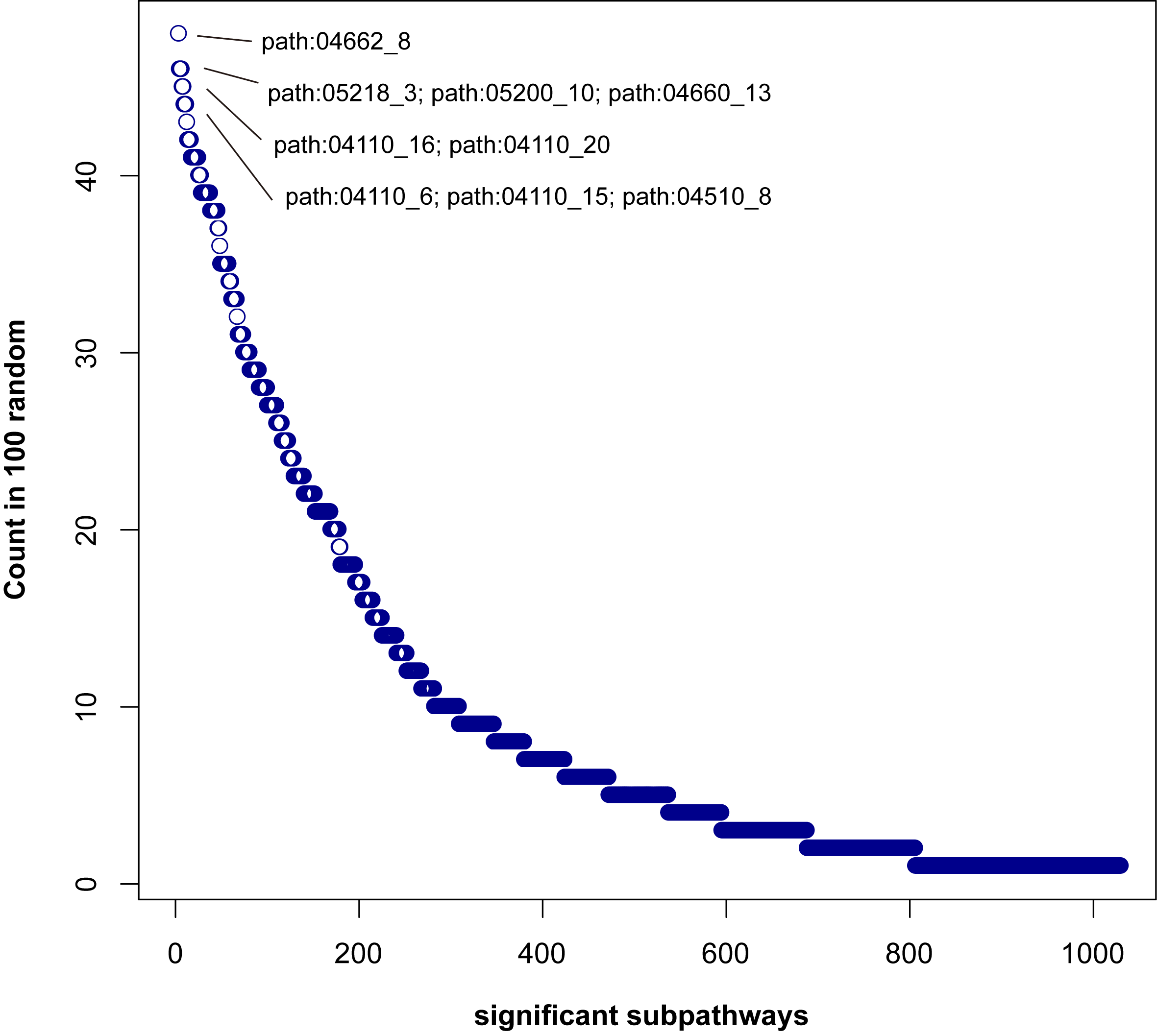

Supplement: S1 Fig — (TIF) [file pone.0194245.s001.tif]

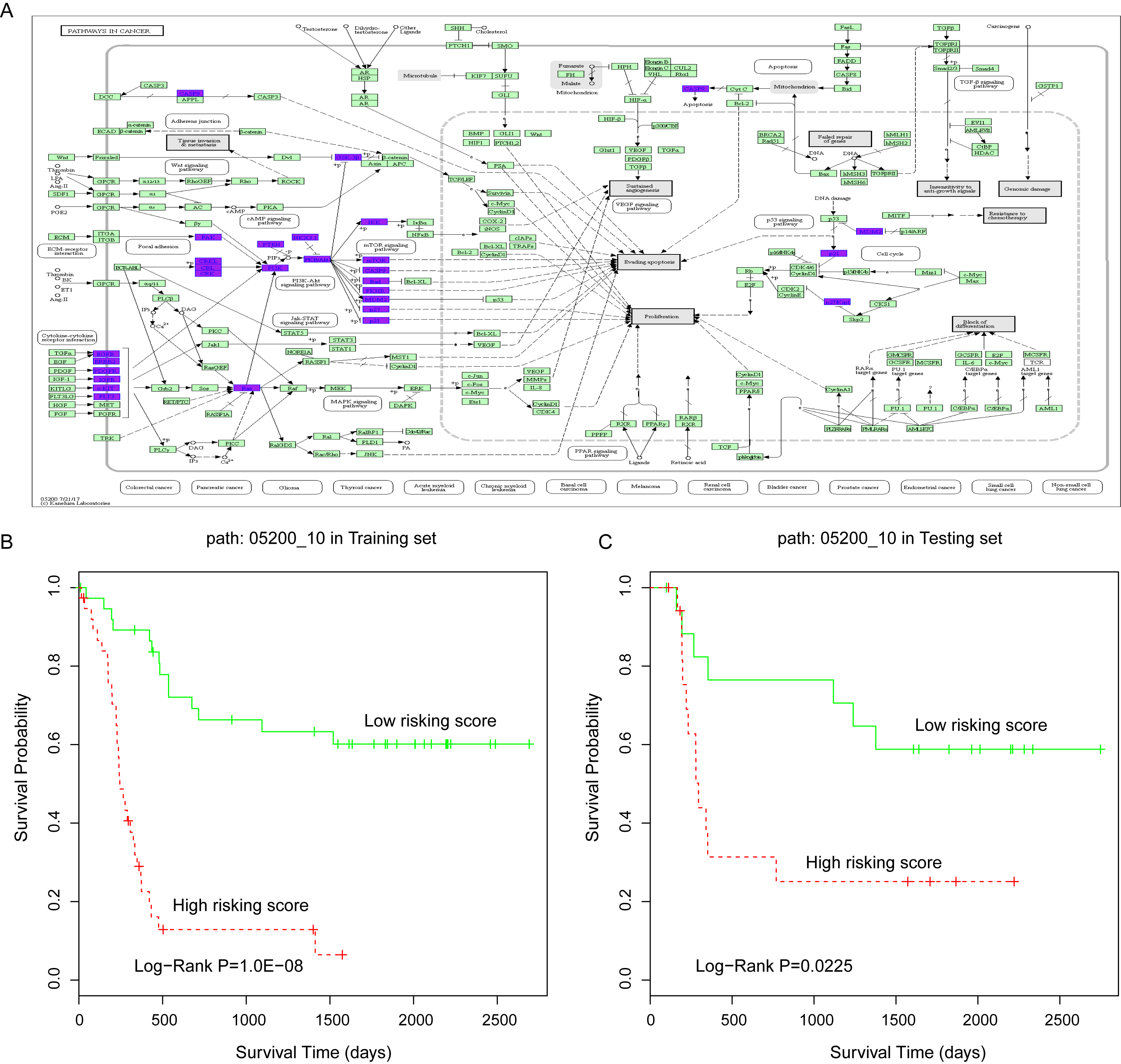

Supplement: S2 Fig — (A) Subpathway_10 from Pathways in cancer (path: 05200). (B) The 05200_10 in the training set. (C) The 05200_10 in the testing set. (TIF) [file pone.0194245.s002.tif]
