# Supplementary material for: Identification of a robust subpathway-based signature for acute myeloid leukemia prognosis using an miRNA integrated strategy
Source: PLoS One. 2018 Mar 23;13(3):e0194245. doi: 10.1371/journal.pone.0194245 (PMC5865743; doi:10.1371/journal.pone.0194245)
Supplement: S2 Table — (DOC) [file pone.0194245.s004.doc]

**Table S2.** **Clinicopathological characteristics of patients in the training set, the testing set, and entire patient set.**

| **Characteristic** | **Training set (N=76)** | **Testing set (N=36)** | **Entire patient set (N=112)** |
| --- | --- | --- | --- |
| **Gender** |  |  |  |
| **Male (%)** | 39 (51.3%) | 17 (47.2%) | 56 (50.0%) |
| **Female (%)** | 37 (48.7%) | 19 (52.8%) | 56 (50.0%) |
| **Patient survival** |  |  |  |
| **Alive (%)** | 40 (52.6%) | 18 (50.0%) | 58 (51.8%) |
| **Deceased (%)** | 36 (47.4%) | 18 (50.0%) | 54 (48.2%) |
| **Survival days (Mean±SD)** | 1546.4±1137.2 | 1568.1±1027.0 | 1553.2±1099.0 |
